# Multidisciplinary investigation reveals the earliest textiles and cinnabar-coloured cloth in Iberian Peninsula

**DOI:** 10.1038/s41598-021-01349-5

**Published:** 2021-11-09

**Authors:** Margarita Gleba, M. Dolores Bretones-García, Corrado Cimarelli, Juan Carlos Vera-Rodríguez, Rafael M. Martínez-Sánchez

**Affiliations:** 1 Department of Cultural Heritage, Università Degli Studi di Padova, Piazza Capitaniato 7, 35139 Padova, Italy; 2 Territorial Delegation of Cultural Heritage, C/Martínez-Montañés 8, 23007 Jaén, Spain; 3 Department of Earth and Natural Sciences, Ludwig-Maximilians-Universität München, Theresienstrasse 41, 80333 Munich, Germany; 4 Department of History, Geography and Anthropology, Universidad de Huelva, Campus de El Carmen, Avda. de las Fuerzas Armadas, s/n, 21071 Huelva, Spain; 5 Department of History, Universidad de Córdoba, P/Hospital Cardenal Salazar 3, 14071 Córdoba, Spain

**Keywords:** Imaging, Mass spectrometry, Microscopy, Archaeology, Geology

## Abstract

Textile production is among the most fundamental and more complex technologies in human prehistory, but is under-investigated due to the perishable nature of fibrous materials. Here we report a discovery of five textile fragments from a prehistoric (fourth-third millennium cal BC) burial deposit located in a small cave at Peñacalera in Sierra Morena hills, near Córdoba, Southern Spain. These textiles accompanied a set of human remains as grave goods, together with other organic elements such as fragments of wood and cork, and some pottery vessels. They were characterized and dated using digital microscopy, Scanning Electron Microscopy, Energy Dispersive Spectroscopy and Accelerator Mass Spectrometry. Two of the fragments described here are the oldest examples of loom-woven textiles in the Iberian Peninsula, dating from the second half of the fourth millennium cal BC. This correlates chronologically with the first appearance of loom weights in the archaeological record of this region. The more recently dated textile is the earliest preserved cloth intentionally coloured with cinnabar in the western Mediterranean. The Peñacalera finds are a key reference for understanding the development of textile technologies during the Neolithic and Copper Age in western Europe and beyond.

## Introduction

Textiles have been an essential element of human material culture since at least the Neolithic period, but their poor preservation often hampers our understanding of the development of this fundamental technology. The discovery of uncharred textile or basketry remains, preserved by desiccation, is particularly rare in archaeological contexts of the Western Mediterranean, and finds dating to the Neolithic period and Copper Age are exceptional. In the Iberian Peninsula, these finds are usually concentrated in the south-eastern region, which currently has a sub-arid climate^1^. Among the best-known cases, are the outstanding Neolithic examples of basketwork made of esparto grass and wooden objects from the Los Murciélagos cave in Albuñol (Granada), dated to fifth millennium cal BC^2,3^, and textiles from the site of Los Millares (Almeria) dated two millennia later^4^. Examples of cordage, esparto basketry and linen textiles are also known from various Bronze Age contexts of El Argar culture (c. 2200 cal BC and 1550 cal BC) in Almería, Murcia and Alicante^4–6^. Among the most unique prehistoric Iberian textile finds are the remains of two linen tunics found in the so-called Cueva Sagrada I (Lorca, Murcia), alongside other organic materials such as esparto and wood, and the remains of five individuals, dated c. 2300 cal BC^7–9^.

Here, we present five textile fragments dated to the Late Neolithic and Copper Age (fourth-third millennium cal BC), found in a burial context inside a small cave in a rocky outcrop located in the Province of Córdoba, in the southwest of Spain. The cave was discovered at the end of 2014 by speleologists, who immediately recognised the significance of the find. In March 2016, an archaeological intervention was carried out (directed by M.D.B.G.), which included full planimetry of the cavity's space and stratigraphic excavation.

**Figure 1:**
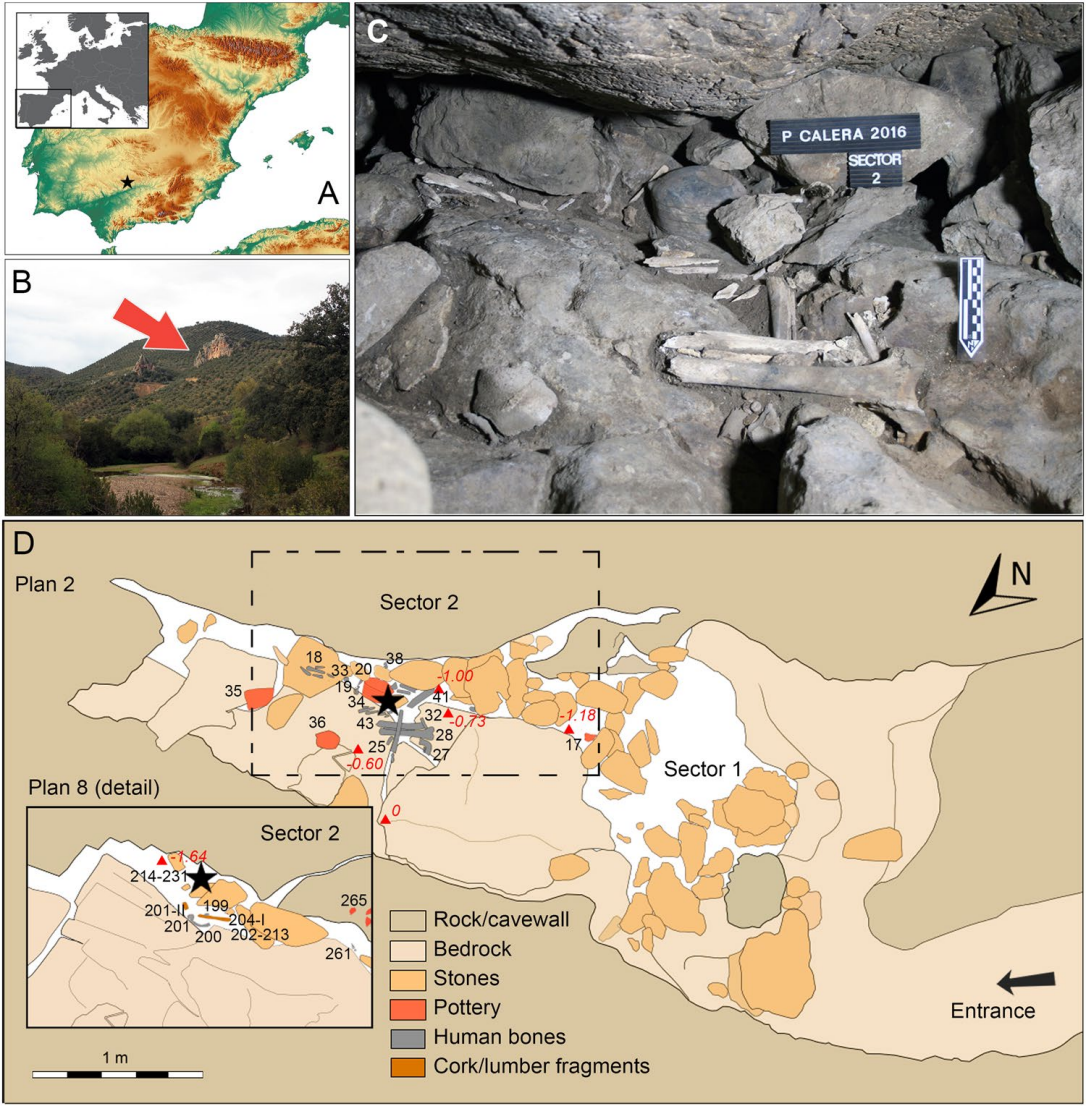
(**A**) Location of the Peñacalera site in the south of the Iberian Peninsula. (**B**) Guadalbarbo Creek Valley, with limestone outcrops behind, where the cavity is located, marked with a red arrow. (**C**) Image of the burial context of Peñacalera, sector 2, where Textile 1 was found. (**D**) Plan 2 of the burial context with detailed Plan 8; red numbers indicate relative depths, black numbers are inventory numbers of the finds; the black star in Plan 2 indicates the approximate location of Textile 5’s find spot; the black star in Plan 8 indicates the find spot of Textile 1. Maps generated by Inkscape (https://inkscape.org/).

The so-called Cueva de la Peña de La Calera (or Peñacalera Cave) is a small cavity formed by a fracture oriented W–E, located in one of the rocky outcrops of the so-called Cerro de La Calera, which is situated southwest of the village of Obejo (Córdoba, Andalusia, Spain), next to the river bed of the Guadalbarbo stream, in the Guadalquivir River basin (38°6′19.24″N, 4°49′56.13″W) (Fig. 1A,B).

The archaeological remains were concentrated under the east wall, scattered amongst the stone blocks of different sizes that formed a kind of cairn up to 1 m thick (Fig. 1C). This deposit was to some extent altered by post-depositional processes, such as percolation and bioturbation, small carnivores having used the space as a den. Underneath and among the stone blocks, were the disarticulated bones of a minimum of five human individuals and various burial goods. The latter consisted of at least eight ceramic vessels, a bone awl, a lithic polisher, and various fragments of cork tree bark (Fig. 1D). Together with these materials, five textile fragments were recovered. All of the fragments were found in sector 2, in a very small space and at a short distance from each other. One of the fragments (PC16 S-2 215-I, hereafter Textile 1) was found during excavation and was associated with two complete ceramic vessels deposited together and directly related to highly fragmented human remains (Fig. 1D Plan 8). Fragment PC16 S-2 34-I (hereafter Textile 5) was recovered with a sample of vegetal turf material excavated together with a ceramic vessel (34) and subsequently identified as a textile (Fig. 1D Plan 2). The other three fragments (PC16 S-2 C-TEX-1A, PC16 S-2 C-TEX-1B, PC16 S-2 C-ESP-3, hereafter Textiles 2, 3 and 4), were recovered during manual sieving of the sediment, and were isolated once they had been identified as textiles. The spatial data collected during the excavation revealed that Textile 5 was recovered from a shallow depth, in the most superficial stone layer of the assemblage. Textile 1 was found in the same sector, but at a depth of approximately 0.64 m with respect to the level of Textile 5.

**Figure 2:**
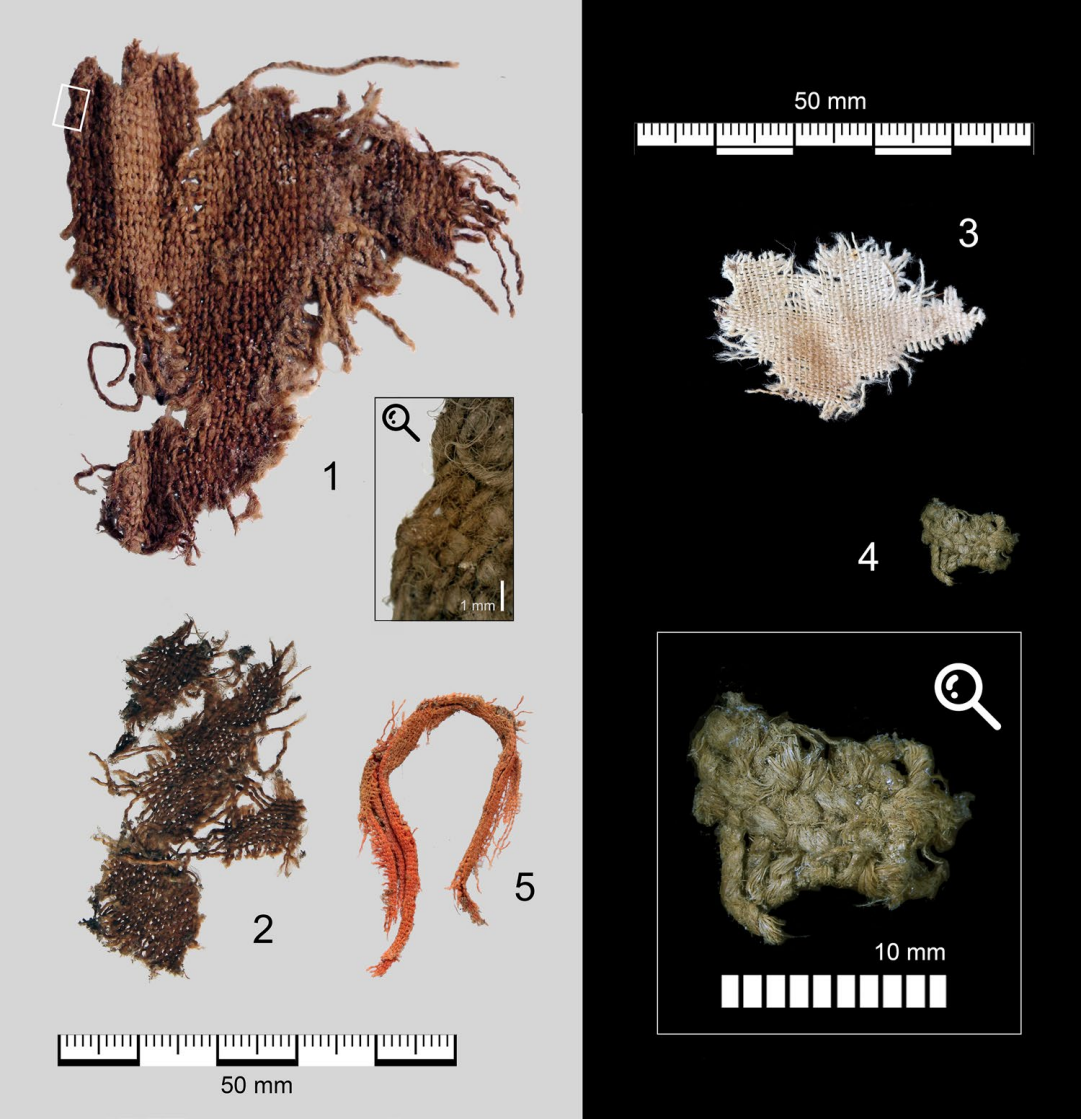
Textiles 1–5 recovered from Pañacalera at the same scale. For technical details of all fragments, see Table 1.

The five textile fragments (Fig. 2) were preserved by desiccation in the stable and dry environment of the cave and are still organic, with much of their original texture, colour and elasticity intact. These are finds of great importance, since direct evidence of the prehistoric textile production in the region has, until now, not been attested before the third millennium cal BC. The finds were characterized using digital microscopy for structural textile analysis, Scanning Electron Microscopy (SEM) for fibre determination, Energy Dispersive Spectroscopy (EDS) for the chemical characterization of the colourant. Four of the textiles were dated using Accelerator Mass Spectrometry (AMS) radiocarbon dating. The results of this multidisciplinary investigation are presented below.

**Table 1:** Textile structural data summary for the five textiles from Peñacalera and (in italics) other Copper and Bonze Age Iberian textile finds (data for Lorca from^8,9^; for Los Millares, El Argar and El Oficio from^4,5^).

Object	Weave	Warp count	Weft count	Warp twist	Weft twist	Warp diameter	Weft diameter
Pañacalera textile 1 (Inv. PC16 S-2 215-I)	Warp-faced plain weave	15	7	Z2*s	Z2*s	0.7–0.8	0.6–1
Pañacalera textile 2 (Inv.PC16 S-2 C-TEX-1A)	Warp-dominant plain weave	18–20	12	Z2*i	Z2*i	0.3–0.4	0.4–0.6
Pañacalera textile 3 (Inv. PC16 S-2 C-TEX-1B)	Warp-faced plain weave	28–30	14–16	Z2*i	Z2*i	0.2–0.3	0.2–0.4
Pañacalera textile 4 (Inv. PC16 S-2 C-ESP-3)	Balanced plain weave	6	6	Z2*s	Z2*s	0.8–0.9	0.9–1
Pañacalera textile 5 (Inv. PC16 S-2 34-I)	Warp-faced plain weave	43–45	21–22	Z2*i	Z2*i	0.1–0.3	0.1–0.3
*Lorca tunic A*	*Warp-dominant plain weave*	*12–18*	*9–15*	Z2*i-s	Z2*i-s	*0.3–0.5*	*0.2–0.7*
*Lorca tunic B*	*Weft-dominant plain weave*	*14–16*	*23–24*	Z2*i-s	Z2*i-s	*0.3–0.5*	*0.2–0.4*
*Lorca textile frag. B*	*Weft-dominant plain weave*	*21*	*28*	Z2*i-s	Z2*i-s	*0.1–0.2*	*0.1–0.2*
*Lorca textile frag. C*	*Warp-dominant plain weave*	*20*	*13*	Z2*i-s	Z2*i-s	*0.4–0.7*	*0.4–0.7*
*Los Millares (various burials*	*Plain weave*	*11–14*	*12–16*	Z2*i-s	Z2*i-s	*0.2–0.3*	*0.2–0.3*
*El Argar (various burials)*	*Plain weave*	*8–27*	*8–27*	Z2*i-s	Z2*i-s	*0.2–0.9*	*0.2–0.9*
*El Oficio (various burials)*	*Plain weave*	*8–19*	*8–19*	Z2*i-s	Z2*i-s	*0.3–1*	*0.3–1*

## Results

### Textile characterisation

**Figure 3:**
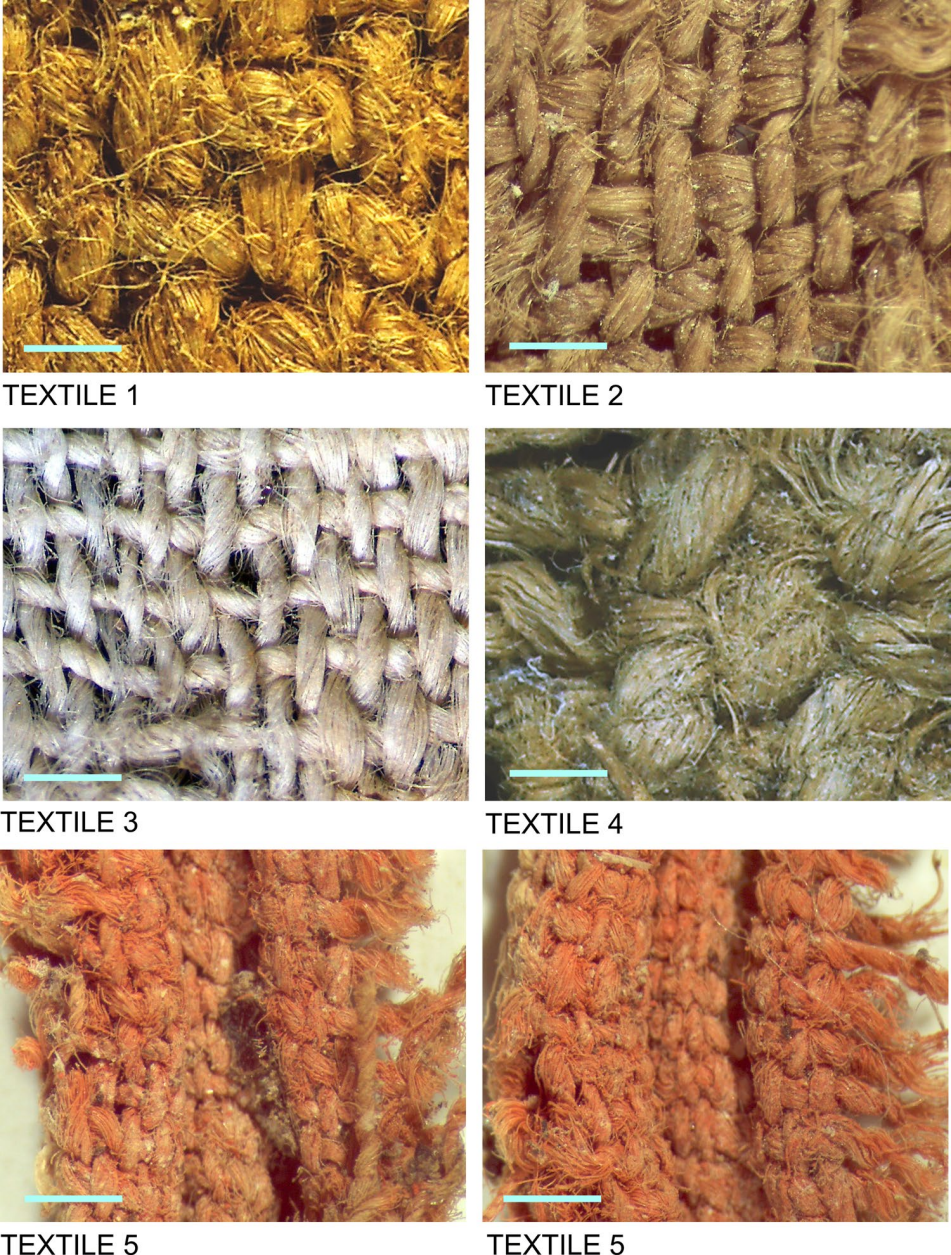
Micrographs of textile weaves. Scale bar in each panel is one millimeter.

All five textiles are woven in plain weave or tabby (Fig. 3). Plain weave is one of the earliest loom-woven structures as it is the simplest textile binding attainable with two thread systems on a loom, with passive warp and active weft threads alternating one over one in each direction. Textile 4 is a balanced plain weave, with approximately the same number of threads per unit of length in warp and weft. Textile 1 is a warp-faced plain weave, with twice as many warps as wefts per unit of length; the warp direction is indicated by the small area of preserved simple selvedge (see detail in Fig. 2(1)). In the selvedge, the weft turns every fourth thread, possibly indicating the use of two wefts during weaving. The remaining three textiles are likely warp-dominant or warp-faced as well, although they do not preserve any selvedges or borders for a definitive identification of warp and weft systems.

Based on the thread counts and thread diameters (Fig. 3 and Table 1), the textiles can be divided into three groups, with two coarser (Textiles 1 and 4), two finer (Textiles 2 and 3) and one exceptionally fine fabric (Textile 5). With 43–45 warp threads and 21–22 weft threads per cm, the latter is the finest prehistoric fabric discovered to date in the Iberian Peninsula, as demonstrated by comparative structural data from textiles discovered at other Copper and Bronze Age sites (section in italics of Table 1).

**Figure 4:**
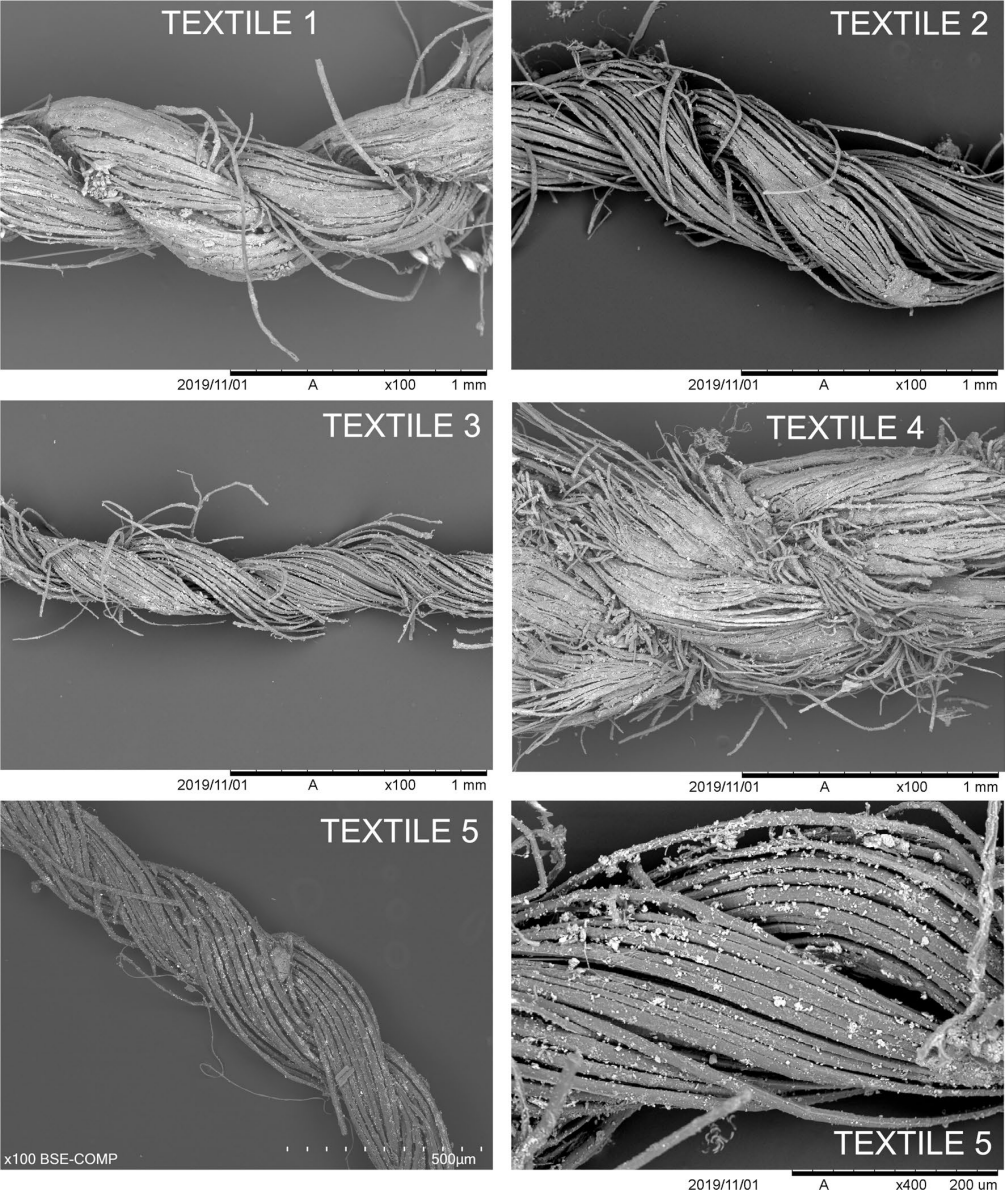
SEM micrographs of the threads showing the spliced nature; bottom right image shows a closeup of fibre bundles.

The five textiles are woven in spliced rather than draft-spun yarn (Fig. 4). Splicing is a technique used to convert plant fibre into yarn that, until recently, has been assumed to have been used exclusively in ancient Egypt and East Asia^10,11^. In contrast to draft spinning, during which the combed and prepared fibres are fixed on a distaff and are continuously drawn to receive a twist through the rotation of a spindle, in splicing, the ends of pre-formed fibre bundles stripped from plant stalks are joined, so that the ends of the fibres would overlap in bunches. Splicing has recently been identified in Neolithic, Bronze and Iron Age textiles in Italy, Greece, Spain and more widely across Europe and western Asia^12^. All of the prehistoric woven linen textiles found in Spain to date appear to be made using spliced yarn.

In the two coarser fragments (Textiles 1 and 4), the single threads have a clear counterclockwise- or s-twist, while in the other three fragments no twist is discernible in the single threads, or only a very faint s-twist. This may be a chronological difference as observed in Egypt^10^, since Textiles 1 and 4 are the oldest (see below on Dating). Yarn for the earlier textiles was likely made using continuous splicing, while the latter appears to have end-to-end splices^12^, although the fragments are too small to identify the splicing technique definitively.

### Fibre characterisation

All five textiles are made of plant bast (stem) fibres, which are present in ultimates bundles (Fig. 4 bottom right), and in Textile 4 also preserve remains of epidermal and parenchymal tissues. The fibres display nodes/dislocations and polygonal cross-sections characteristic of plant bast fibre (Supplementary Material Table 1 and Fig. 1). The cracks in the fibres of Textiles 3 and 5 are S or counterclockwise, possibly indicating s-microfibrillar orientation. Fibre diameter measurements range between 6.9 and 25.9 µm and mean diameters in the five fragments are between 13.1 and 19.7 µm. All the diagnostic features are consistent with flax (*Linum* sp.)^13^.

### Colourant characterisation

**Figure 5:**
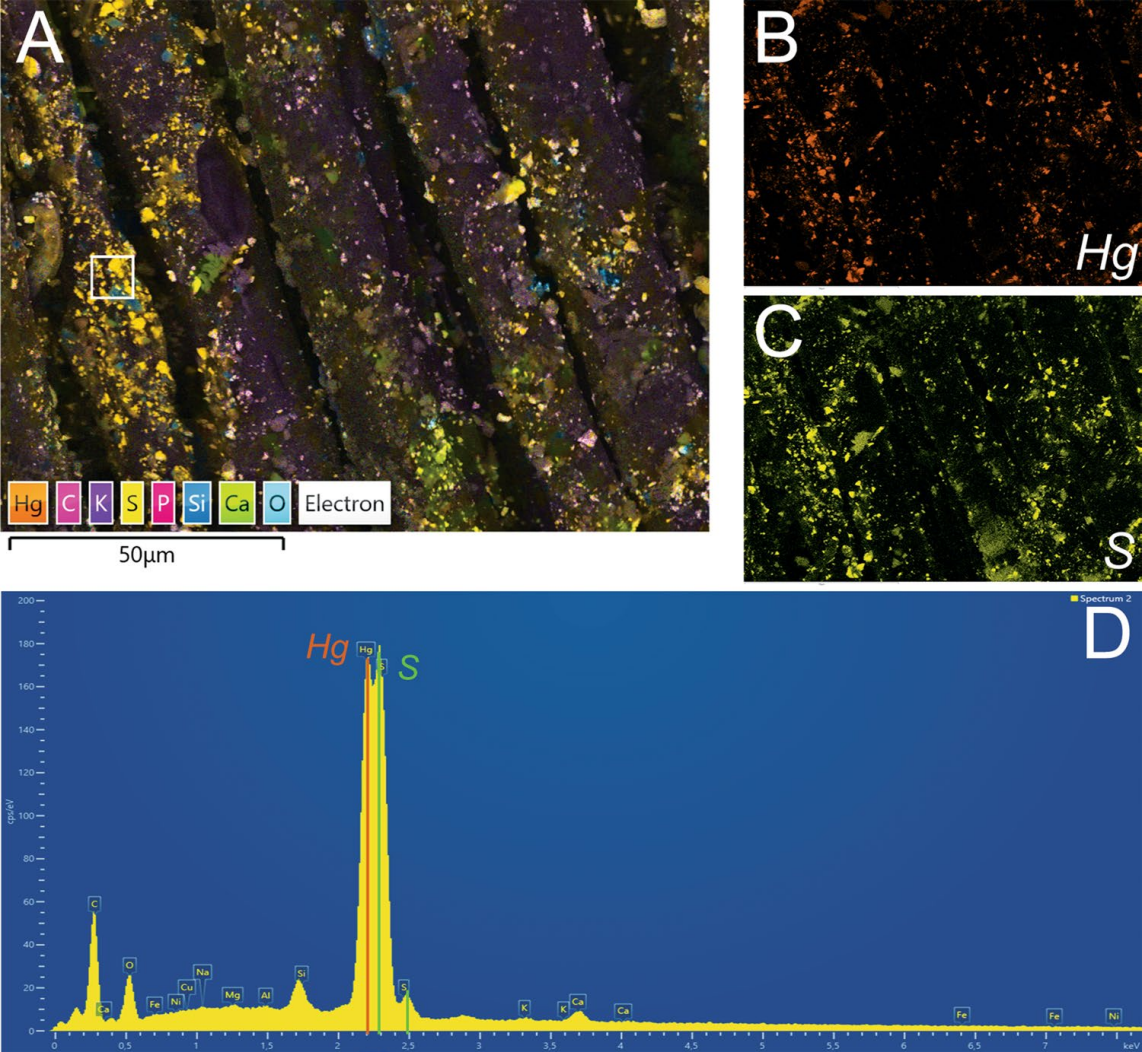
Elemental composition analysis of warp sample from Textile 5: (**A**) composite EDS elemental map; (**B**) EDS elemental map of Hg (mercury); (**C**) EDS elemental map of S (sulphur); (**D**) spectrum of the composite EDS map.

Elemental composition analysis of warp and weft samples from Textile 5, which has a distinct and homogeneously reddish pink colour unequivocally showed the spatial association of clear peaks of Hg (mercury) and S (sulphur), which are elements of the red mineral cinnabar (mercury sulphide, HgS). This association of elements is evident both on EDS maps of the whole sample (Fig. 5A–D), as well as single-point spectra executed locally on the portion of the samples where the mineralization was more consistent (see Supplementary Material Fig. 2). No traces of cinnabar were found on the other four textile samples (see Supplementary Material Fig. 3).

### Dating

**Figure 6:**
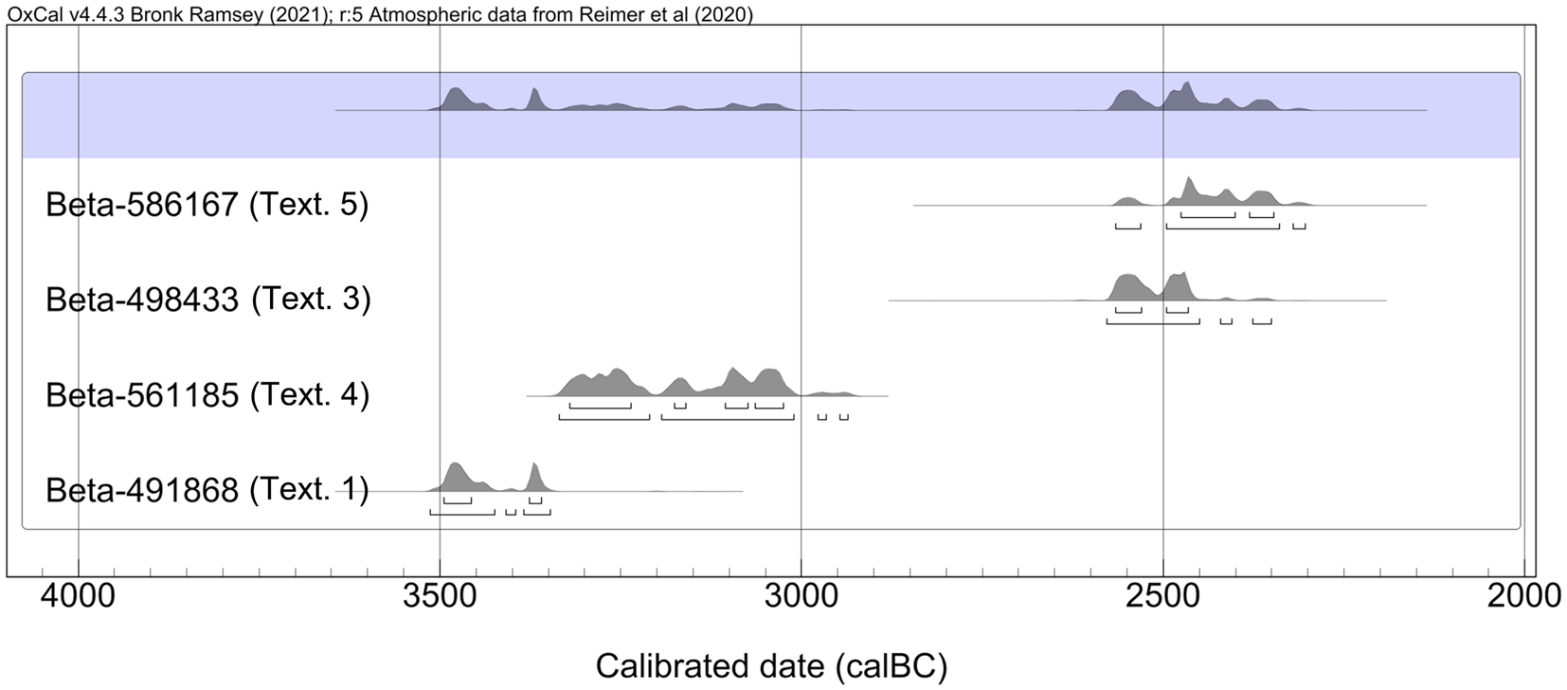
Multi-plot with the AMS dates obtained on four of the textiles. On top, sum of probabilities. Calibrated from the IntCal20 curve^14^. For details, see Supplementary Material Table 2.

Direct dating of four of the five textiles by radiocarbon-AMS resulted in them grouping into two distinct chronological ranges (Fig. 6; Supplementary Material Table 2). The earlier pertains to Textiles 1 and 4. Both dates (Beta-491868, 4620 ± 30 BP and Beta-561185, 4450 ± 30 BP) are circumscribed within the second half of the fourth millennium cal BC, which also fits with a well defined pottery typology of some of the grave goods found in the cave. The dates pertaining to the earliest period of cave use thus point to a mean chronology of calibrated dates (IntCal20 atmospheric curve) of c. 3400 cal BC. The dating of the finer Textiles 3 (Beta-498433, 3980 ± 33 BP) and 5 (Beta-586167, 3940 ± 30 BP), yielded an average of c. 2500–2300 cal BC, being almost a millennium later than the other two dated textiles.

## Discussion

The textile finds from Peñacalera are important in terms of their raw materials, thread structure, production technique and decoration (use of colour), since they are the earliest examples known to date of loom-woven and cinnabar-coloured textiles from the Iberian Peninsula.

Flax is believed to have been introduced to the Iberian Peninsula as part of the Neolithic package^5,15^. Before its introduction no similar bast fibres appear to have been used in the region. Until now, the earliest linen textiles known in Spain were found in the Copper Age contexts of southeastern Iberia, such as the site of Los Millares (3200–2300 BC) and other contemporary settlements^4^, the Cueva Sagrada I at Lorca^8,9^, as well as the various Bronze Age cemeteries of the Argaric Culture^4,5^. The textiles from Peñacalera are thus significantly older, pushing the date for the use of linen in textiles back in time and making Textile 1 the oldest loom-woven textile in Spain made of flax fibre. As such, it provides the direct evidence of flax use for linen textile production in Iberian Peninsula during the Late Neolithic period and indicates that flax was introduced as a fibre crop by this point in time.

Like other analysed Copper and Early Bronze Age linen textiles found in Iberian Peninsula, the textiles of Peñacalera are woven with spliced yarn, which is in agreement with contemporaneous plant fibre textiles across Europe, Egypt and western Asia^12^. The use of splicing may also be indirectly indicated by the rarity or almost complete absence of spindle whorls in the Neolithic and Copper Age period in southern Iberia^16^, although they could have been made in perishable materials and did not survive.

The regular plain weave structure of all textiles and the partly preserved selvedge of Textile 1 from Peñacalera indicate that they were woven on a loom, thus constituting the earliest direct evidence of this technology in the Iberian Peninsula. Until the discovery of these textiles, the earliest evidence for the use of this technology were the third-millennium cal BC tunics of Cueva Sagrada 1, which preserve a fringed finishing border, typical for loom-woven textiles^9^. In Central Europe, particularly in the Alpine lake dwellings in Switzerland, loom-woven linen textiles began to appear from the second quarter of the fourth millennium cal BC^17,18^, just a few centuries before the earliest Peñacalera textile.

The earliest archaeologically-attested looms in Iberia and more widely across Europe were of the vertical warp-weighted type, which utilizes loom weights to put tension on the warp threads^19,20^. Having frequently been made of fired clay, loom weights survive well in the archaeological record and serve as proxies for the use of the warp-weighted loom technology. The earliest evidence for the use of the warp-weighted loom comes from Central Europe and dates to the late sixth millennium BC^21^. In southern Iberia, loom weights appear by the middle of the fourth millennium cal BC, shortly before the appearance of the so-called “Carinated Bowls Complex”, which marks the beginning of the Copper Age in the region^22^. In the second half of the fourth and during the third millennium cal BC, the loom weights are of crescent or rectangular plaque shape, with perforations at the ends^23^. They are ubiquitous in settlement sites, sometimes found in groups attesting their use in sets^24^. Slightly flattened crescents, with vertical perforations at the ends, have been found for example at La Loma (Illora, Granada)^25^, Casa del Tabaco (El Carpio, Córdoba)^26^, while a later variant with horizontal perforations constitutes an important element of the material culture in many settlements of southwestern Iberia. They are abundant in Perdigões (Alentejo, Portugal)^27^, São Pedro (Redondo, Portugal)^28^, Castillejos de Montefrío (Granada)^29^, and Polideportivo-La Alberquilla (Martos, Jaén)^30^ throughout the second half of the fourth millennium BC, and continue to be used in this region during the third millennium BC. The earliest loom weight evidence in southern Iberia is thus contemporaneous with the earliest Peñacalera textile.

Cinnabar is a natural mineral of volcanic or hydrothermal origin composed of mercury and sulphur (HgS), with a characteristic scarlet-red colour. It has been mined in Europe, Asia and the Americas to be used as pigment for pottery decoration and body paint, in burials, metallurgy and for medicinal purposes. Bands of cinnabar are present on a Pre-Pottery Neolithic B (ninth-eighth millennium BC) child skull at Tel Abu Hureira in Syria^31^ and a woman’s skull at Çatalhöyük in Turkey, where cinnabar was used not only in burial contexts but also as pigment in wall paintings^32^. Almadén de la Plata (Ciudad Real) in central Spain is one of the largest natural sources of cinnabar in the world^33^, and analytical studies have demonstrated this site as one of the main sources of cinnabar present at other archaeological sites^34^. The exploitation and use of cinnabar as a pigment in Iberian prehistory is a well documented phenomenon. It has been mined with certainty since Classical antiquity, when this exploitation is referenced in written sources, until today^35^, but a recent study indicates that the use of the cinnabar deriving from the region during the Late Neolithic and Copper Age period in Portugal was sufficiently extensive to cause mild to severe mercury poisoning in the prehistoric population^36^.

The earliest evidence of human use of cinnabar in Iberia dates back to the Early Neolithic (5500–4800 cal BC), when its use as a colouring material to fill impressions and incisions on ceramic vessels and stone bracelets is documented at Cueva de Los Murciélagos (Zuheros, Córdoba)^37^. Cinnabar powder was found inside a *Glycymeris* shell container in Cova de l'Or (Beniarrés, Alicante), dated to the same period^38^, while a flint blade covered with cinnabar has been reported in the Casa Montero flint mine (Madrid), exploited between 5300–5100 cal BC^39^. Red soils, resulting from the presence of powdered cinnabar, have been documented on the chamber floors of some Andalusian megalithic monuments, such as Alberite (Villamartín, Cádiz)^40^ and the menhir of Casas de Don Pedro (Belmez, Córdoba)^41^, both dated to the fifth millennium cal BC. From more recent times (first half of the fourth millennium cal BC), it is reported in the Velilla dolmen (Osorno, Palencia) and in the monuments of southern Portugal, such as Anta Grande do Zambujeiro (Alentejo)^42^.

From the Copper Age onwards (3300–2200 cal BC), its use increased, as demonstrated by the finds at the Dolmen of Casas de Don Pedro in the Guadiato Valley (Córdoba)^43–46^. The cinnabar powder has been found on and under the skeletal remains of numerous funerary deposits, their number rising considerably during the third millennium cal BC. In the Montelirio passage tomb (Castilleja de Guzmán, Seville), dated c. 2800 cal BC, cinnabar is present on various artefacts, on human remains and the chamber stones, sharing space with exotic luxury materials such as ivory, gold, amber, rock crystal and ostrich eggs^44^. In that tomb, and specifically in its so-called Great Chamber, at least 20 individuals have been identified, mostly women, wearing hundreds of thousands of white discoidal beads, interpreted as embellishment of the funerary clothing or textiles^45^. In some cases, cinnabar powder was found covering beads or directly associated with the skeletal remains, a possible evidence of cinnabar-coloured textiles within the same assemblage. In other cases, direct association with human skeletal remains has been explained as body paint^47^.

More than a century ago, Louis Siret hypothesised the presence of textiles coloured with cinnabar in tomb 356 of the El Argar necropolis (2200–1700 cal BC) on the basis of the red bands observed on the skull^48,49^, and textile imprints in clay preserving cinnabar particles^35^. The recently discovered skeletal remains and grave goods documented in the Bell Beaker culture necropolis of Humanejos (Parla, Madrid), display direct impregnation with cinnabar. An interesting case is that of an elderly male (individual 1) from tomb 5, who displays three linear bands of cinnabar transferred directly onto the skull, indicating original presence of cinnabar-coloured textile bands or headwear decorated with linear motifs. Furthermore, alongside the face of this individual, a large cinnabar stain was identified, which has been interpreted as evidence of a bag or textile coloured with or containing cinnabar^50^.

Despite the continuing discussion regarding the original use of cinnabar as either body paint, a ritual burial element or textile colourant, until now there has been no direct evidence of cinnabar use to colour textiles in prehistoric Iberia. The discovery of cinnabar on Textile 5 from Peñacalera cave provides the first and earliest unequivocal proof of the use of this substance as a textile colourant at least by the Copper Age in the Iberian Peninsula. The geological ambiance of the Peñacalera cave is sedimentary limestones of the Namurian (Carboniferous), a rock of marine origin with fossils of crinoids, with no possibility of the natural presence of cinnabar. Since the mineral is absent from the other textiles and on any other objects found in the cave, and the textile is completely impregnated with the mineral powder, it could not have been coloured by transfer; rather we suggest it was intentionally coloured with the mineral. Cinnabar is not a dye which chemically binds to fibre but a mineral pigment. The method of applying it to textiles remains unknown but clearly involves grinding it to a fine powder and its physical application to the textile surface either by rubbing or soaking it in a suspension.

Although small and fragmentary, the textile finds from Peñacalera add important new information to our understanding of the development of textile technologies in the Iberian Peninsula and Europe during the Late Neolithic period and Copper Age. By the second half of the fourth millennium cal BC, loom-woven textiles were being used by the south Iberian populations, as also indirectly confirmed by the loom weight evidence. The thread was produced using splicing technique, which was refined over time allowing to produce exceptionally fine threads and textiles by the middle of the third millennium cal BC. Also by this time, the use of cinnabar to colour textiles was well developed as demonstrated by the extremely homogeneous distribution of the mineral on Textile 5.

## Methods

### Textile structural analysis

The structural analysis was carried out using autopic observation, portable Dino-Lite AM7115MZT digital microscope at different magnifications (× 20, × 50, × 230) and a binocular microscope Leica M80 with optical magnifications until × 60, with built-in-camera EC3 Hd, focus × 0.5, in the Laboratory of Archaeometry A. Arribas-Palau, University of Granada, Spain. Textile analysis involved determination of structural parameters such as weave and thread count per cm, thread twist, diameter and angle, presence of edges and any other diagnostic features^51^. Due to the small size of Textile 5, warp thread count was calculated based on the count carried out in several areas 2.5 mm wide.

### Fibre identification and analysis

Fibre identification was carried out using Hitachi TM3000 TableTop Scanning Electron Microscope at the McDonald Institute for Archaeological Research, University of Cambridge, UK (Textiles 1–4) and Hitachi SU 5000 at the Ludwig Maximilian University of Munich, Germany (Textile 5). The samples were analysed to determine the morphological characteristics of the fibre and to acquire more detailed surface information for fibre species identification. The following instrumental settings were used: variable vacuum conditions, analytical condition mode at 15.00 kV accelerating voltage, compositional imaging and working distance of 5–10 mm. The samples were not coated. The observed features were compared with M.G.’s fibre reference collection. The diameter of fibres was measured using the SEM utility tool at × 400 magnification.

### Elemental composition analysis

All textiles and a sample of sediment from the cave were analysed using a Scanning Electron Microscope equipped with an Energy Dispersive Spectrometry analyser (SEM–EDS) at the Department of Earth and Natural Sciences at the Ludwig Maximilian University of Munich. The EDS sensor uses a Ultim®Max Silicon Drift Detector (SDD) produced by Oxford Instruments, which allows chemical and elemental point and map analysis of the sample. The analysis of the samples has been conducted in variable vacuum condition using an acceleration voltage of 15 kV and a current intensity of 0.14 mA. Both chemical EDS maps and single-point EDS spectra have been collected for the characterization of the samples.

### Radiocarbon dating

Four samples of fibres from Textiles 1, 3, 4 and 5 were radiocarbon dated by Beta Analytic Inc. in Miami, Florida, USA. Due to financial constrains and technical similarity with Textile 3, Textile 2 was not dated. The amounts analysed were 2.5 mg for Textile 1, 0.39 for Textile 3, 2.3 for Textile 4 and, 1.1 for Textile 5. No coating products, preservatives or hardener of any type were used on the remains. All samples were simply pretreated with a cleaning solution of acid/ alkali/ acid before dating. The samples were at first gently crushed then dispersed in deionized water. They were then washed with hot HCl acid to eliminate carbonates, followed by an alkali wash (NaOH) to remove secondary organic acids. The alkali wash was followed by a final acid rinse to neutralize the solution before drying. Each chemical solution was neutralized before the application of the next. During these serial rinses, mechanical contaminants such as associated sediments, organic residues, fulvic acid and rootlets were eliminated.

## Supplementary Information

Supplementary Information.

## Data Availability

All data generated or analysed during this study are included in the published article and its Supplementary Material file.
