# Supplementary material for: Multidisciplinary investigation reveals the earliest textiles and cinnabar-coloured cloth in Iberian Peninsula
Source: Sci Rep. 2021 Nov 9;11:21918. doi: 10.1038/s41598-021-01349-5 (PMC8578544; doi:10.1038/s41598-021-01349-5)
Supplement: Supplementary file 1 — Supplementary Information. [file 41598_2021_1349_MOESM1_ESM.pdf]

Supplementary Material

Multidisciplinary investigation reveals the earliest textiles and cinnabar-coloured cloth in Iberian Peninsula

Margarita Gleba[1]* ORCID: 0000-0001-7729-7795

M. Dolores Bretones García[2] ORCID: 0000-0003-4941-0310

Corrado Cimarelli[3] ORCID: 0000-0002-5707-5930

Juan Carlos Vera-Rodríguez[4] ORCID: 0000-0002-5989-2694

Rafael M Martínez-Sánchez[5] ORCID: 0000-0002-8947-117X

[1]Department of Cultural History, Università degli Studi di Padova, Piazza Capitaniato 7, 35139 Padova, Italy

[2] Territorial Delegation of Cultural Heritage, C/ Martínez-Montañés 5, 23007 Jaén, Spain

[3]Department of Earth and Natural Sciences, Ludwig-Maximilians-Universität München, Theresienstrasse 41, 80333, Munich, Germany

[4]Department of History, Geography and Anthropology, Universidad de Huelva, Campus de El Carmen, Avda. de las Fuerzas Armadas, s/n 21071-Huelva, Spain

[5]Department of History, Universidad de Córdoba, P/ Hospital Cardenal Salazar 3, 14071, Córdoba, Spain

*Corresponding Author: margarita.gleba@unipd.it

Object	No of fibres	Mean diameter (micron)	Diameter range (micron)	Characteristics	Material
Textile 1	7	19.7	15.8–24.2	fibre bundles;<br>weak nodes/dislocations	plant bast, likely flax (*Linum* sp.)
Textile 2	25	16.7	9.6–25.9	fibre bundles;<br>polygonal cross section;<br>nodes/dislocations	plant bast, likely flax (*Linum* sp.)
Textile 3	31	13.1	6.9–19.2	fibre bundles;<br>polygonal cross section;<br>nodes/dislocations;<br>S-splitting of fibres;<br>small epidermis fragments	plant bast, flax (*Linum* sp.)
Textile 4	14	13.3	5.6–23.2	fibre bundles;<br>polygonal cross section;<br>nodes/dislocations	plant bast, likely flax (*Linum* sp.)
Textile 5	6	15.8	12.4–20.1	fibre bundles;<br>S-splitting of fibres;<br>nodes/dislocations	plant bast, likely flax (*Linum* sp.)

Supplementary Table 1. Fibre characteristics and identification of Peñacalera textiles.

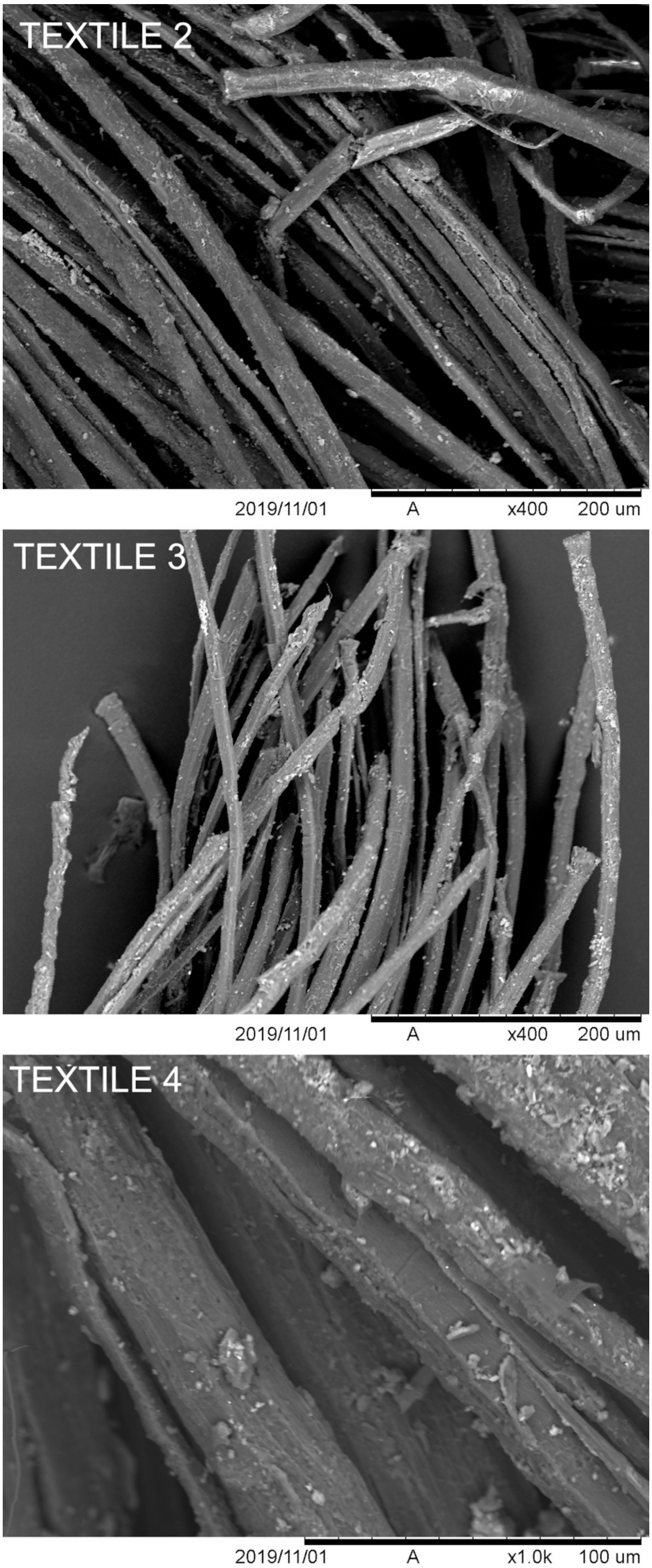

Supplementary Figure 1. SEM micrographs of fibres in Peñacalera textiles showing flax characteristics.

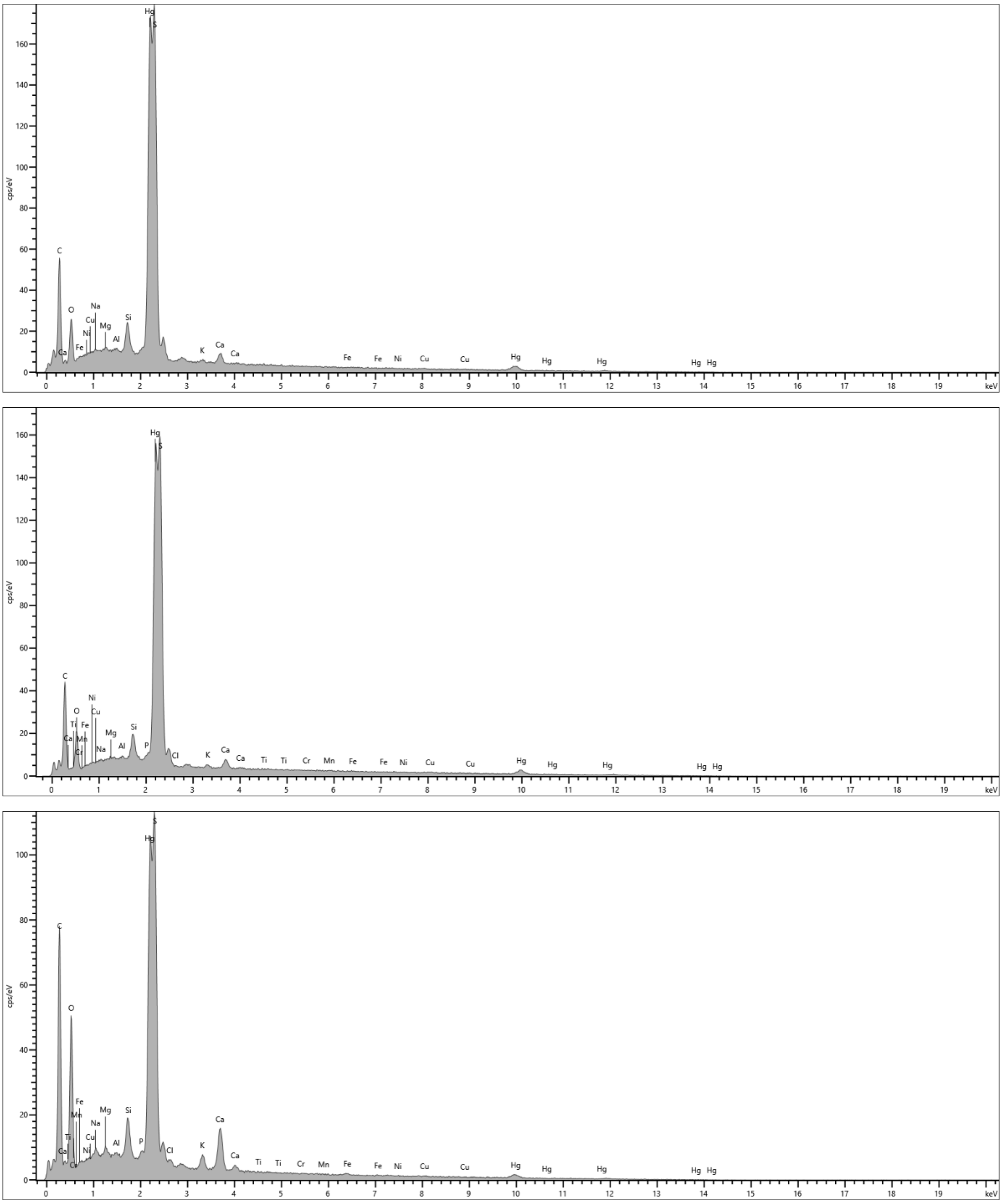

Supplementary Figure 2. Elemental composition analysis of warp sample from Textile 5 from Peñacalera: Single-point spectra executed locally on the portions where the mineral presence is more consistent.

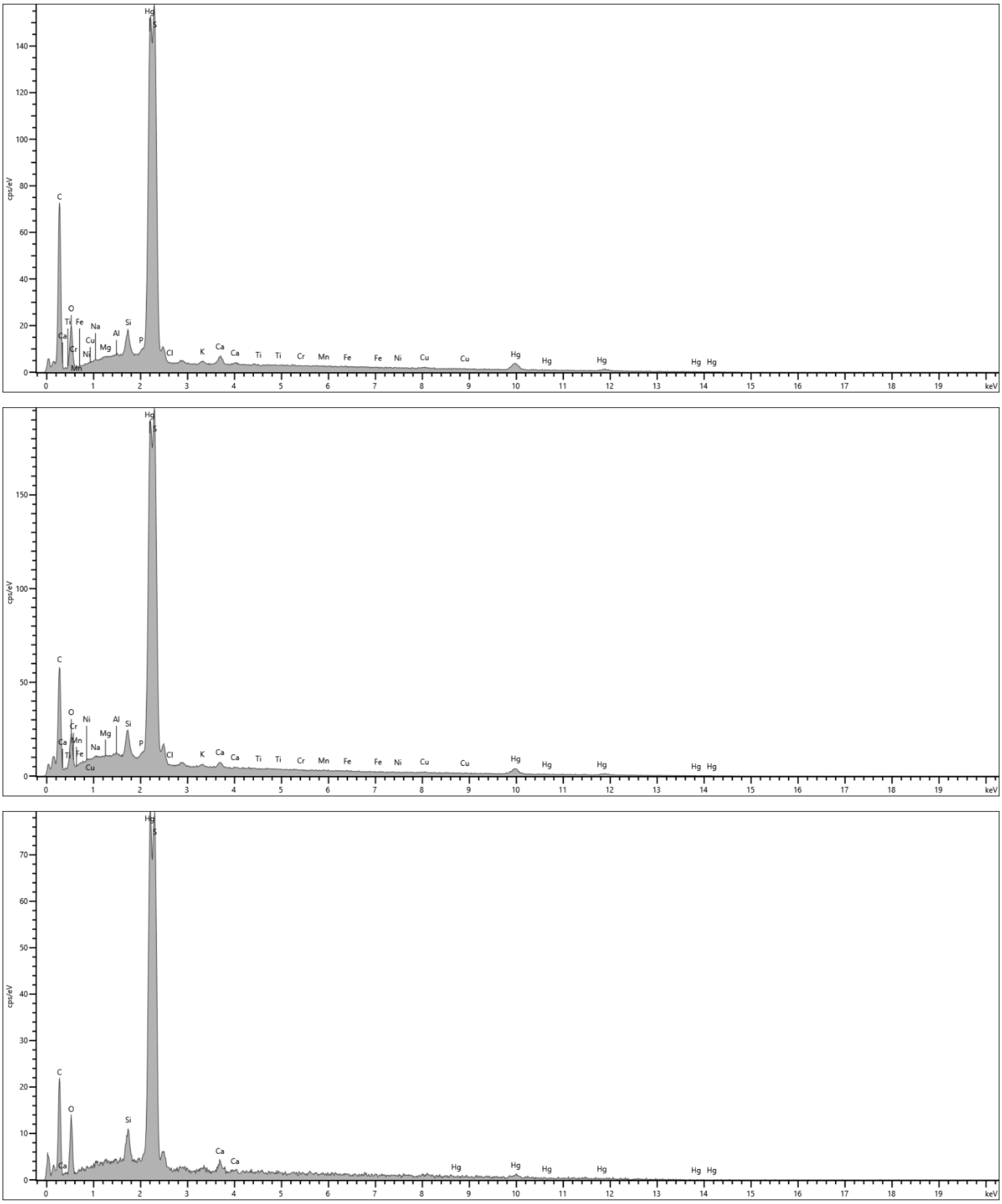

Supplementary Figure 3 (continued). Elemental composition analysis of warp sample from Textile 5 from Peñacalera: Single-point spectra executed locally on the portions where the mineral presence is more consistent.

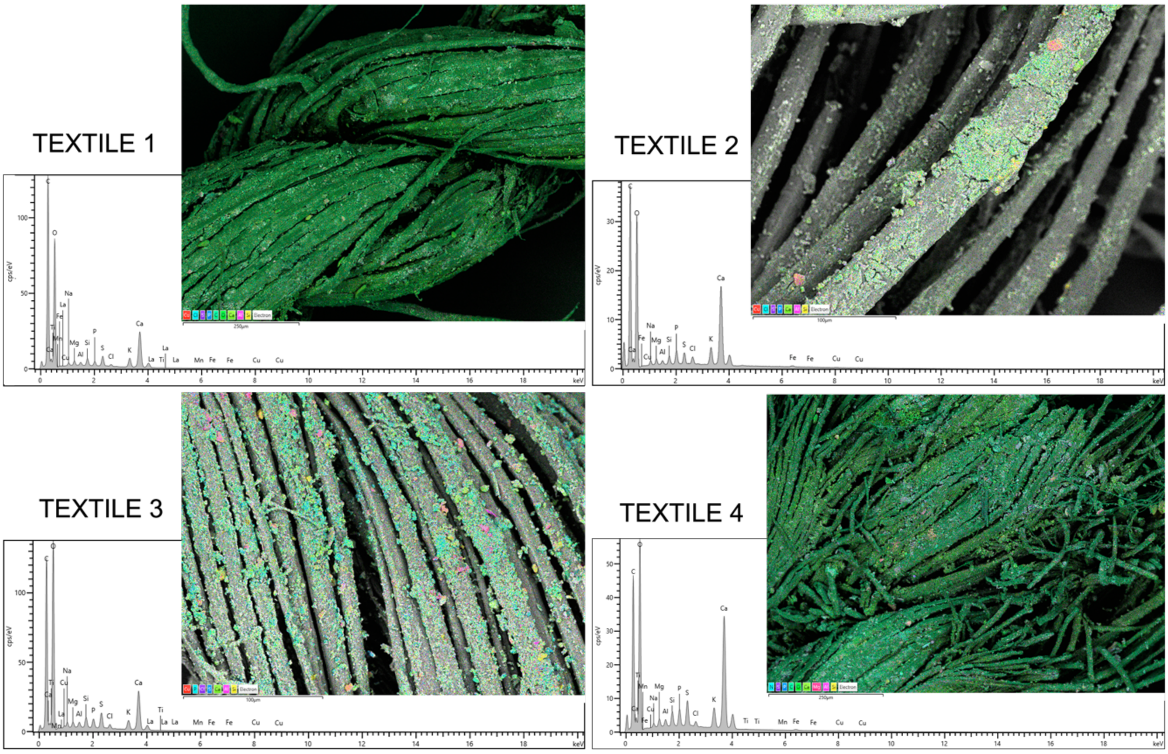

Supplementary Figure 3. Elemental composition analysis of sediment and Textiles 1-4 from Peñacalera.

**ID**	**CODE**	**BP**	**SD**	**Cal BC 68.3 %**	**Cal BC 95.4 %**	**M cal BC**	**‰$\delta^{13}C$**
Textile 1	Beta-491868	4620	30	3495-3360	3514-3348	3462	-24.5
Textile 4	Beta-561185	4450	30	3321-3026	3336-2937	3163	-23.4
Textile 3	Beta-498433	3980	33	2567-2466	2578-2351	2515	-25.6
Textile 5	Beta-586167	3940	30	2476-2348	2566-2305	2432	-25.5

Supplementary Table 2. Numerical values of the AMS dates obtained on textiles from Peñacalera. Calibrated from the IntCal20 curve [14].
